# Discovery of a 2′-Fluoro,2′-Bromouridine Phosphoramidate Prodrug Exhibiting Anti-Yellow Fever Virus Activity in Culture and in Mice

**DOI:** 10.3390/microorganisms10112098

**Published:** 2022-10-22

**Authors:** Julia C. LeCher, Keivan Zandi, Vivian Vasconcelos Costa, Franck Amblard, Sijia Tao, Dharmeshkumar Patel, Sujin Lee, Felipe Rocha da Silva Santos, Matheus Rodrigues Goncalves, Celso Martins Queroz-Junior, Fernanda Martins Marim, Katie Musall, Shu Ling Goh, Tamara McBrayer, Jessica Downs-Bowen, Ramyani De, Niloufar Azadi, James Kohler, Mauro Martins Teixeira, Raymond F. Schinazi

**Affiliations:** 1 Center for ViroScience and Cure, Laboratory of Biochemical Pharmacology, Department of Pediatrics, Emory University School of Medicine, Atlanta, GA 30322, USA; 2 Department of Morphology, Instituto de Ciências Biológicas, Universidade Federal de Minas Gerais, Belo Horizonte 31270-901, Brazil; 3 Department of Biochemistry and Immunology, Instituto de Ciências Biológicas, Universidade Federal de Minas Gerais, Belo Horizonte 31270-901, Brazil

**Keywords:** flavivirus, yellow fever virus, nucleoside analogs, antiviral agents, A129 mouse model

## Abstract

Yellow fever virus (YFV) is a potentially lethal, zoonotic, blood-borne flavivirus transmitted to humans and non-human primates by mosquitoes. Owing to multiple deadly epidemics, the WHO classifies YFV as a “high impact, high threat disease” with resurgent epidemic potential. At present, there are no approved antiviral therapies to combat YFV infection. Herein we report on 2′-halogen-modified nucleoside analogs as potential anti-YFV agents. Of 11 compounds evaluated, three showed great promise with low toxicity, high intracellular metabolism into the active nucleoside triphosphate form, and sub-micromolar anti-YFV activity. Notably, we investigated a 2′-fluoro,2′-bromouridine phosphate prodrug (C9), a known anti-HCV agent with good stability in human blood and favorable metabolism. Predictive modeling revealed that C9 could readily bind the active site of the YFV RdRp, conferring its anti-YFV activity. C9 displayed potent anti-YFV activity in primary human macrophages, 3D hepatocyte spheroids, and in mice. In an A129 murine model, shortly after infection, C9 significantly reduced YFV replication and protected against YFV-induced liver inflammation and pathology with no adverse effects. Collectively, this work identifies a potent new anti-YFV agent with strong therapeutic promise.

## 1. Introduction

Yellow fever virus (YFV) is a blood-borne, zoonotic, human, and non-human primate (NHP) pathogen of the family *Flaviviridae* transmitted via mosquito vector (*Aedes aegypti* sp., *Haemagogus leucocelaenus* sp.) endemic in tropical and subtropical regions of Africa and South America [1,2,3]. Infections range from mild to life-threatening with death occurring in ~39% of cases [2,3,4]. Following a mosquito bite, the virus infects sub-dermal Langerhans before being transmitted to circulating macrophages of the lymph, which then carry the virus to different organ systems [5]. The liver is the primary target organ for YFV, resulting in jaundice, from which the virus derives its name, and over 50% of lethal cases show hepatic involvement [2,3,4]. Late-phase hemorrhaging and organ failure are the primary causes of YFV-related deaths [2,3,4].

Attempts to eradicate YFV are made difficult by the fact that non-human primates serve as viral reservoirs, maintaining the virus through constant transmission between vector and host. Currently, there are no antiviral agents to treat YFV infection though effort has been made to develop new or repurpose other flavivirus-specific antiviral agents to combat YFV. Nucleoside analogs (NA) are an attractive class of antiviral agents as they target the viral RNA-dependent RNA polymerase (RdRp) and can exhibit potent antiviral activity with minimal cytotoxicity [6,7]. Our team and others reported that sofosbuvir, a 2′-modified halogen nucleoside prodrug approved for the treatment of hepatitis C (HCV), has potent anti-YFV activity in vitro [8,9,10,11]. Unfortunately, in mouse model, sofosbuvir offered protection against YFV infection only when given prophylactically [10].

**Figure 1:**
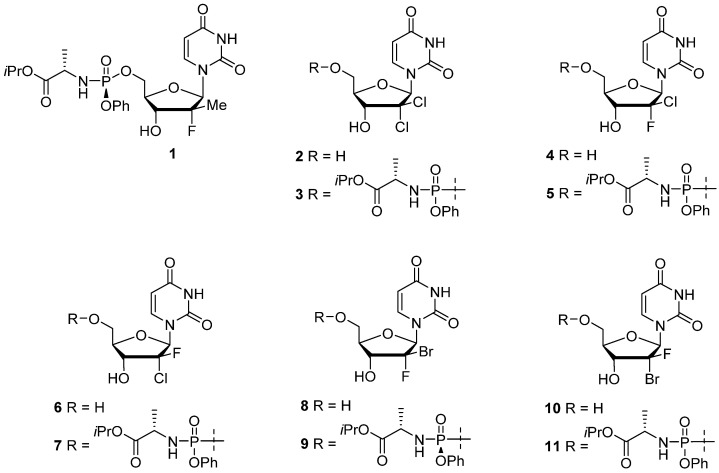
Structures of sofosbuvir (1) and 2′-dihalonucleosides and phosphoramidate prodrugs 2-11.

In this study, a series of 2′-dihalogen-substituted uridine NAs and their respective prodrugs, including known HCV RdRp inhibitors [12,13,14], were evaluated for anti-YFV activity. As the liver is a primary YFV target, we first evaluated our 11 compounds in human hepatoma cells. Our data confirmed the reported in vitro anti-YFV activity of both sofosbuvir and compound C3 (Figure 1; [11]) as well as newly identifying the anti-YFV activity of two anti-HCV compounds, a 2′-chloro,2′-fluorouridine (C5 [12]) and a 2′-fluoro,2′-bromouridine (C9 [13]). C9 was further assessed in a 3D hepatoma spheroid model and in primary human macrophages as well as in the YFV A129 mouse model. In 2D and 3D hepatoma cultures and primary macrophages, C9 exhibited sub-to-low micromolar anti-YFV activity and low cytotoxicity. In mice, C9 reduced levels of serum transaminase and provided protection to the liver with a significant reduction in virus burden, inflammation, and steatosis. These results are notable as they demonstrate the identification of an anti-HCV [13] nucleoside analog that also has potent activity against YFV both in vitro and in vivo and provide a framework for its preclinical development.

## 2. Materials and Methods

### 2.1. Cells, Virus, and Media

Human hepatocarcinoma liver epithelial cells (Huh-7; JCRB0403) were purchased from JCRB Cell Bank, JCRB. African Green Monkey Kidney cells (Vero; ATCC^®^ CCL-81TM) and human lymphoblasts (CEM; ATCC^®^ CRL-2265TM) were purchased from ATCC (Manassas, VA, USA). Primary human peripheral blood mononuclear (PBM) cells and primary human peripheral blood monocyte-derived macrophages (MDM) were isolated in-house from human blood obtained from NY Blood Bank (New York, NY, USA) as previously described [15,16,17]. MDMs were generated from PBM cells isolated from blood buffy coats from healthy donors (NY Blood Bank, New York, NY, USA) via Ficoll-Hypaque density gradient centrifugation. After isolation, PBM cells were washed with phosphate-buffered saline (PBS) and resuspended in complete RPMI 10 media (Roswell Park Memorial Institute) (RPMI) 1640 Medium, 10% fetal calf serum (FBS), and 100 U/100 µg/mL penicillin/streptomycin (pen/strep). Next, monocytes were isolated by CD14 positive selection using autoMACS buffer (1 mL 0.5 M EDTA + 37.5 mL liquid bovine serum albumin (BSA) + 461.5 mL PBS) according to the manufacturer’s protocol (Miltenyi Biotech, Bergisch Gladbach, Germany). For MDM differentiation, purified CD14^+^ monocytes were cultured in complete RPMI 10 media with 7 ng/mL granulocyte–macrophage colony-stimulating factor (GM-CSF) (Miltenyi Biotech, Germany) for at least 8 days. Primary human hepatocytes were purchased from BioIVT (Westbury, NY, USA). Media compositions were as follows: (1) Huh-7—Dulbecco’s modified eagle medium (DMEM), 10% FBS, 100 U/mL pen-strep, and 2 µM L-glutamine (L-glut), (2) Vero—DMEM, 10% FBS, 100 U/mL pen-strep, and 2 µM L-glut, (3) CEM—RPMI 1640 Medium, 10% FBS, and 100 U/100 µg/mL pen/strep, (4, 5) PBM, MDM- RPMI 1640 Medium, 10% FBS, 100 IU/mL IL-2, and 100 U/100 µg/mL pen/strep, (6) primary human hepatocytes—InVitroGRO HI Medium with 2.2% Antibiotic Mix Torpedo (BioIVT, Westbury, NY). For all experiments, cells were grown at 37 °C in a 95% O_2_, 5% CO_2_ incubator. The live attenuated YFV strain 17D was propagated in-house in Huh-7 cells as previously described [18] to generate working virus stocks with a titer of 10^8^ PFU/mL.

### 2.2. Synthesis of Compounds

Nucleoside derivatives (designated as compounds C1-11) were synthesized in our laboratory according to previously reported procedures [12,13,19,20] and were determined to be at least 98% pure by liquid chromatography-mass spectrometry (LC-MS) and nuclear magnetic resonance spectroscopy (NMR). Chemical structures for these compounds are summarized in Figure 1.

### 2.3. 3D Hepatoma Spheroid Generation

Human hepatoma spheroids (HHOs) were generated from Huh-7 cells over 7 days in a 1:5 slab matrix of Matrigel^®^ Basement Membrane Matrix (Corning, NY, USA) diluted with DMEM. In brief, 50 µL of the diluted matrix was plated in each well of a low-attachment spheroid 96-well microplate and incubated at 37 °C in a 95% O_2_, 5% CO_2_ incubator overnight. The next day, 10^3^ cells in 50 µL DMEM containing 10% FBS (10% DMEM) per well were seeded on top of the matrix for a total FBS concentration of 5% per well. HHOs were fed every 3 days with the addition of 50 µL of 10% DMEM. HHOs were routinely assessed for spheroid formation and diameter by light microscopy (EVOS^®^ FL Cell Imaging System; Advanced Microscopy, Bothell, WA, USA) and viability by trypan dye exclusion assay (VI-CELL XR; Beckman Coulter, Indianapolis, IN, USA). After 10 days in culture, healthy spheroids (>80% viability) with a diameter of ~80–100 µm were used for indicated assays.

### 2.4. MTS Cytotoxicity Assay

Cellular toxicity of compounds was assayed as previously described [17] for Vero, Huh-7, CEM, PBM, and MDM cells by their ability to metabolize 3-(4,5-dimethylthiazol-2-yl)-5-(3-carboxymethoxyphenyl)-2-(4-sulfophenyl)-2H-tetrazolium (MTS) [21] in the presence or absence of increasing concentrations of compounds (0–100 µM). Briefly, 10^3^ (Vero, Huh7) or 10^5^ (CEM, PBM, MDM) cells were seeded into 96-well plates in respective media except for MDMs which were kept in RPMI 1640 Medium, 10% FBS, 100 IU/mL IL-2, 0.1% sodium bicarbonate, and 100 U/100 µg/mL pen/strep. Cells were treated with 1, 10, or 100 µM of the compound for 4 days. For controls, cells were treated with 0.1, 1.0, or 10 µM of cycloheximide (CHX) or left untreated. After 3 (Vero), 4 (CEM, Huh-7, MDMs), or 5 (PBM cells) days, 15 µL (Vero, Huh-7, CEM, PBM cells) or 20 µL (MDM) of MTS reagent (Promega©, Madison, WI, USA) was added per well and optical density reads were taken after 1–4 hr at 490 nm absorbance on a multi-mode plate reader (Synergy, BioTek^®^, Winooski, VT, USA). Plain media controls were used to subtract background absorbance and CC_50_ values were calculated using the Chou and Talalay method [22]. MTS assays were performed in spheroids similarly with the following modifications: (1) After 10 days in culture, spheroids were treated with 0, 1, 10, or 100 µM of compounds or 0.1, 1.0, or 10 µM of CHX in full media and incubated for 4 days, (2) 20 µL of MTS reagent was added per well, and (3) reads were taken at 4 hr after treatment. The utility of the MTS assay with spheroids was validated by comparison to the standard trypan blue dye exclusion assay.

### 2.5. Antiviral Evaluation Assay

For antiviral evaluation, (1) Huh-7 cells were grown to confluency (10^4^ cells) or (2) MDMs (10^5^ cells pooled from three donors per experiment) were plated and differentiated in 96-well plates. Dose-response curves were performed by 2-fold serial dilutions (0–20 µM) of the compound in respective base media containing 2% heat-inactivated FBS. Treated cells were infected with YFV MOI 0.1 (Huh-7) or 1.0 (MDMs) for 72 hr in an equal volume of media for a final compound concentration of 0–10 µM. Supernatants (Huh-7, MDMs) and cells (MDMs) were collected in 150 µL RLT Buffer (Qiagen©, Hilden, Germany) for RNA extraction (RNeasy 96 extraction kit; Qiagen©, Hilden, Germany) and subsequent qRT-PCR to detect viral load. All 2D antiviral assays were performed three independent times in triplicate (*n* = 9). Advanced antiviral assays were also performed by dose-response curve with lead compounds in 3D hepatocyte spheroids. After 1 week in culture, compounds (0–10 µM final concentration) were added to spheroids along with YFV (MOI 1.0) and infections proceeded for 3 days. After 3 days, RNA was isolated from cultures using a high-through-put methodology developed by our group. In brief, supernatants and cells were collected and transferred into a sterile 96-well S-Block (Qiagen©, Hilden, Germany). Culture wells were rinsed 3× with 1× phosphate-buffered saline (PBS), trypsinized to collect adherent cells, and trypsin inactivated with an equal volume of FBS. The resultant trypsin/FBS mixture was transferred to spheroids/matrix in S-Block and cells disassociated via repeated pipetting and then pelleted by centrifugation (500× *g*, 5 min). Supernatants were removed and transferred to a new S-block while cells were washed with PBS, resuspended, and pelleted. After 2× repeats, cells were resuspended and lysed in 150 µL RLT Buffer (Qiagen©, Hilden, Germany) for downstream RNA extraction (RNeasy 96 extraction kit; Qiagen©, Hilden, Germany) and subsequent qRT-PCR to detect viral load. All 3D-based assays were performed three independent times in duplicate (*n* = 6).

### 2.6. qRT-PCR

Virus yield inhibition assays were performed as previously described [11]. In brief, viral RNA was detected by real-time PCR using a 6-carboxyfluorescein (FAM)-labeled probe with primers against YFV (forward primer, 5′- AGA AGA TTG GTT AGA TGA TGA TAG T-3′; reverse primer, 5′- TTC CAT CTC TAA TTG AGG TTG AAC C-3′; and probe, 5′-56-FAM/TC CTC ACT GCC GTC TTG TTG ACC A/3BHQ_1-3′) (IDT™, Newark, NJ, USA). RNA isolated from uninfected cells served as a negative control. RNA was added to optimized 10 µM primer/probe mix in Mastermix (qScript™ XLT One-Step RT-qPCR ToughMix^®^; Quantabio, Beverly, MA, USA) and run on a Lightcycler^®^ 480 II (Roche, Germany) according to the manufacturer’s protocol. C_T_ values were calculated from replicate groups then virus yield was quantified via a standard curve. The median effective concentration of compounds (EC_50_) and concentrations with a 90% inhibitory effect (EC_90_) were calculated using GraphPad Prism, v 9 (GraphPad Software Inc., San Diego, CA, USA) and reported as the mean ± standard error of the mean (SEM).

### 2.7. Cellular Pharmacology Assay

Cellular pharmacology assays were performed as previously described [23]. In brief, Huh-7 cells or primary hepatocytes were seeded at 10^6^ cells/well in a 12-well cell culture plate and then, after 24 hr, incubated with 50 µM of the compound for 4 hr. Cells were washed twice with ice-cold PBS, scrapped, and pelleted (500× *g*, 5 min). Then, cell pellets were resuspended in 1 mL of 70% methanol and analyzed by LC-MS analysis as previously described [24]. All assays were performed in triplicate (*n* = 3). Results were plotted using GraphPad Prism, v9 (GraphPad Software Inc., San Diego, CA, USA).

### 2.8. Molecular Modeling

The crystal structure of YFV RdRp (PDB ID: 6QSN) was selected. As there is no NTP in the YFV RdRP active site pocket, NTP was transferred from highly homologous (79% sequence similarity) Japanese encephalitis RdRp (PDB ID: 4HDH). The bioactive conformation of ATP was transferred to YFV RdRp through Maestro of Schrödinger suite. A nucleic acid mutator plugin from the Schrödinger suite was used to mutate ATP to UTP. From UTP to 2′-fluoro,2′-methyluridine-5′-triphosphate (parent compound of sofosbuvir/C1) and C8-TP were generated using the Build module of Schrödinger suite. All the structure complexes were minimized and the Prime MM-GBSA module of the Schrödinger suite was used to calculate MMGBSA dG values. For multiple sequence alignment, the protein sequences of the catalytic domain of polymerases from 16 YFV isolates were retrieved from the UniProt database (https://www.uniprot.org/ (accessed on 14 October 2022)). The multiple sequence alignment was performed using ClustalX (https://doi.org/10.1093/bioinformatics/btm404 (accessed on 14 October 2022)) using default parameters and percentage identity was calculated with ClustalX.

### 2.9. In Vivo Infection of A129 Mice with YFV 17D

#### 2.9.1. Ethics Statement

This study was conducted according to the regulations on ethical and animal experiments of the Brazilian Government (law 11794/2008). The experimental protocol was approved by the Committee on Animal Ethics of the Universidade Federal de Minas Gerais (CEUA/UFMG, permit protocol no.84/2018).

#### 2.9.2. Mice and YFV Mouse Infection

In vivo experiments were performed using type I interferon receptor-deficient mice on SV129/Ev background (A129). Male and female A129 mice (8–10 weeks old) were kept under specific pathogen-free conditions at 23 °C on a 12-hr light/12-hr dark cycle with food and water provided *ad libitum* at the Immunopharmacology Laboratory at ICB-UFMG. Mice were inoculated with YFV_17D_ strain by the intravenous (i.v.) route (tail vein) with 10^6^ PFU in a volume of 100 μL phosphate-buffered saline (PBS). Mice were treated intraperitoneally with 10 mg/kg of C9 (200 µL) 1 hr after infection and every 24 hr until day 3. The vehicle group was treated with 200 µL of saline (NaCl 0.9%). Clinical signs of disease and body weight loss were evaluated daily. On day 3 post-YFV infection, mice were euthanized to obtain blood and liver for further analysis. All surgeries were performed under ketamine/xylazine anesthesia. For the studies, six mice were used per group (four groups), with an even mix of male and female mice for a total of 24 mice.

#### 2.9.3. Hematological Analysis

Blood samples were collected from the cava vein of mice using heparin-containing syringes. Total and differential leukocyte counts were determined using the Nihon Kohden’s Celltac MEK-6500K hemocytometer.

#### 2.9.4. Alanine Aminotransferase Measurement

To determine alanine aminotransferase activity (ALT) in serum samples of mice, a kinetic kit (BioClin, Brazil) was used in accordance with the manufacturer’s instructions.

#### 2.9.5. Tissue-Specific Real-Time RT-PCR

For YFV quantification in mouse liver samples, quantitative RT-PCR was performed. First, RNA was isolated using a QIAamp Viral RNA Mini Kit (Qiagen©, Hilden, Germany) and quantified on a NanoDrop (ThermoScientific, Waltham, MA, USA). Total RNA was adjusted to 50 ng/3 µL and submitted to one-step RT-qPCR using the Quantinova Probe RT-PCR Kit (Qiagen©, Hilden, Germany) and the 7500 Fast Real-Time PCR (Applied Biosystems, Waltham, MA, USA). Primers used were: FORWARD: 5′-GCTAATTGAGGTGYATTGGTCTGC-3′; REVERSE: 5′-CTGCTAATCGCTCAAMGAACG-3′; PROBE: 5′-ATCGAGTTGCTAGGCAATAAA CAC-3′.

#### 2.9.6. Histopathological Liver Analysis

Liver samples were stained with hematoxylin and eosin (H and E) and the histopathology score analyses were evaluated according to criteria such as cellular infiltration, hepatocyte swelling, and degeneration, then added to reach a four-point score (0, absent; 1, slight; 2, moderate; 3, marked; and 4, severe) in each analysis. A total of two sections per animal were examined and results were plotted as the mean damage values in each mouse. Image acquisition and analysis were performed using an Olympus BX microscope (Olympus). The images presented are representative.

#### 2.9.7. Statistical Analysis

Means between two groups were compared by student *t* test, and comparisons among groups were analyzed by one-way analysis of variance (ANOVA). A *p*-value of 0.05 or less was considered statistically significant. Data were plotted using GraphPad Prism v8 (GraphPad Software Inc., San Diego, CA, USA).

## 3. Results

Given the established anti-YFV in vitro activity of both sofosbuvir and C3 [9,10,11] as well as previous successes with isoteric replacement of the C2′ hydroxyl groups for potent anti-flavivirus activity [8,12,13,25,26,27,28,29,30,31], we first rescreened and validated previously identified compounds (C3, C5, and C9) then investigated a panel of uridine halogen-modifed NAs and their respective prodrugs (Figure 1). Of note, we initially considered C5 as being inactive since it did not markedly reduce CPE in the rhabdomyosarcoma (RD) skeletal muscle cell line (<2%; [11]). However, the use of a more sensitive qRT-PCR assay demonstrated that this compound was in fact an inhibitor of YFV replication in RD cells (EC_50_ = 0.5 ± 0.2 µM) and thus was included, alongside its parent NA, for further assessment. 

### 3.1. Antiviral and Cytotoxicity of Compounds 1–11

Compounds were assayed for anti-YFV activity by dose-response in Huh-7 cells at 3 days post-infection via qRT-PCR. Sofosbuvir was used as a positive control and showed expected anti-YFV activity with an EC_50_ of 0.6 ± 0.6 µM, consistent with our previous report [11]. Of the 11 compounds screened, parent compounds C2, C4, C6, C8, and C10 did not show marked anti-YFV activity at or below 10 µM, while nucleoside prodrugs C3, C5, C9 demonstrated sub-micromolar anti-YFV activity (EC_50s_ of 0.4 ± 0.5, 0.4 ± 0.2 and 0.9 ± 0.8 µM respectively; Table 1).

Compounds C1-11 were evaluated for cytotoxicity in multiple cell lines representing normal/cancer cells, anchored/non-anchored cells; slow/fast-growing cells, via the ability to metabolize MTS at 4 days post-treatment with 0.1–100 µM of compound (Table 1). As YFV is a blood-borne pathogen and antiviral therapies are typically delivered systemically, compounds were evaluated for cytotoxicity in human PBM cells and lymphoblasts (CEM). As the liver is the primary target for YFV, human hepatomas (Huh-7) were used. The interferon-deficient African Green Monkey Vero cell line, a target cell for YFV and standard in virological studies, was also included. With the exception of C7 and C11, compounds exhibited low-to-no cellular toxicity in all cell systems used (Table 1). Cytotoxicity values for sofosbuvir, C3, C5 [11], and C9 [13] were within the range of previously reported values (Table 1). Notably, prodrugs C7 and C11 were toxic at doses <10 µM in Huh-7s while their respective parents were non-toxic (Table 1).

### 3.2. In Vitro Cellular Metabolism of Lead Compounds

**Figure 2:**
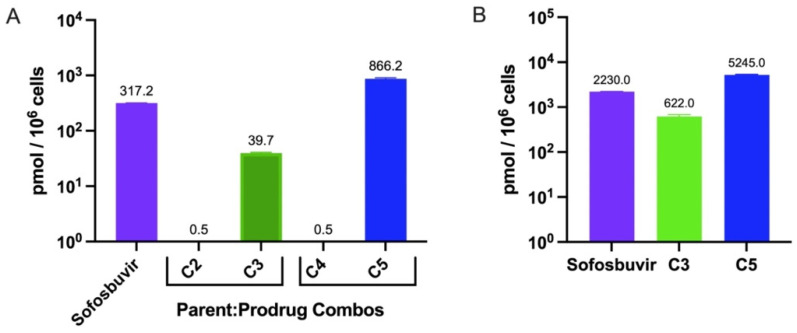
Intracellular metabolism of compounds into active NTP form. Huh-7 cells (**A**) or primary human hepatocytes (**B**) were plated at 10^6^ cells/well and treated with 50 µM of indicated compound for 4 hr then cells were washed and collected for analysis via LC-MS for NTP formation. Data were analyzed by GraphPad Prism v9. Data shown as mean ± SEM, *n* = 3.

NAs require intracellular processing into their active nucleoside triphosphate (NTP) form. To determine levels of NTP conversion and to better understand their mode of action, we assayed phosphorylation levels of the identified hits. As previously established by our group, C9 is readily converted to its NTP form by 4 hr post uptake at 10 µM in Huh-7 cells (646 ± 107 pmol/10^6^ cells) or primary hepatocytes (3206 ± 247 pmol/10^6^ cells) [13]. We assayed the intracellular metabolism of other lead compounds (C3 and C5) alongside their respective parent compounds (C2 and C4) in Huh-7s (Figure 2A) and primary hepatocytes (Figure 2B) via LC-MS. Prodrugs were efficiently metabolized intracellularly into active NTP form within 4 hr of treatment, while treatment with parent compounds produced low levels of NTP by comparison (Figure 2A). Significantly higher levels of NTP were detected in primary hepatocytes compared to Huh-7s (Figure 2). This same trend was noted previously for C9 in primary hepatocytes compared with Huh-7s [13]. Collectively, these data corresponded with noted cellular antiviral activity (Table 1).

### 3.3. Prediction of Binding Mode of C1(Sofosbuvir) and C8/9 Compounds

**Figure 3:**
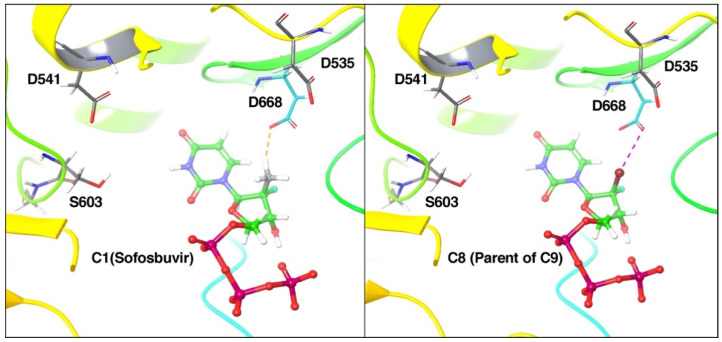
Predicted binding mode of compounds 2′-fluoro,2′-methyluridine-5′-triphosphate, and C8-TP in the active site binding pocket of YFV RdRp. Active site residues are shown in grey sticks while compounds are shown in the ball and stick model. The Orange dashed line represents a steric clash and the violet dashed line represents a halogen bond.

Compound C9 was particularly attractive for further development as it has documented flavivirus activity, presents with low cellular toxicity, has limited specificity for human polymerases, and has favorable pharmacokinetics [12,13]. Our previous work showed that C9 is readily converted into active NTP (C8-TP) in vitro. Using the recently published X-ray crystal structure of YFV RdRp [32], molecular modeling studies were performed to predict and compare the binding mode of 2′-fluoro,2′-methyluridine-5′-triphosphate (parent molecule of sofosbuvir/C1 in TP form) with C8-TP. These studies showed that both NTPs can bind YFV RdRp in a similar way (Figure 3). However, the 2′-bromine in C8-TP is predicted to form a halogen bond with D668 while the hydrophobic 2′-methyl group in 2′-fluoro,2′-methyluridine-5′-triphosphate leads to a steric clash with the same amino acid. As a result, the Prime MM-GBSA dG bind-free energy for C8-TP was −54.11 kcal/mol compared with that of −43.03 kcal/mol for 2′-fluoro,2′-methyluridine-5′-triphosphate (−43.03 kcal/mol). To verify that our predictive binding model would hold for different YFV strains, we performed a multiple sequence alignment of the catalytic domain of 16 YFV strains isolated in Brazil during the 2017–2020 epidemic. Alignment in the active site of the RdRp showed 98.04% homology and conservation in binding site residues, (D535, D541, S603, D668; shown Figure 3) involved in direct interactions with the compound, suggesting that C9′s observed anti-YFV effects will be similar with different YFV strains.

### 3.4. Evaluation of In Vitro Efficacy of Lead Compound C9 for Anti-YFV Activity in 3D Hepatoma Spheroid Model and Primary Human Macrophages

**Figure 4:**
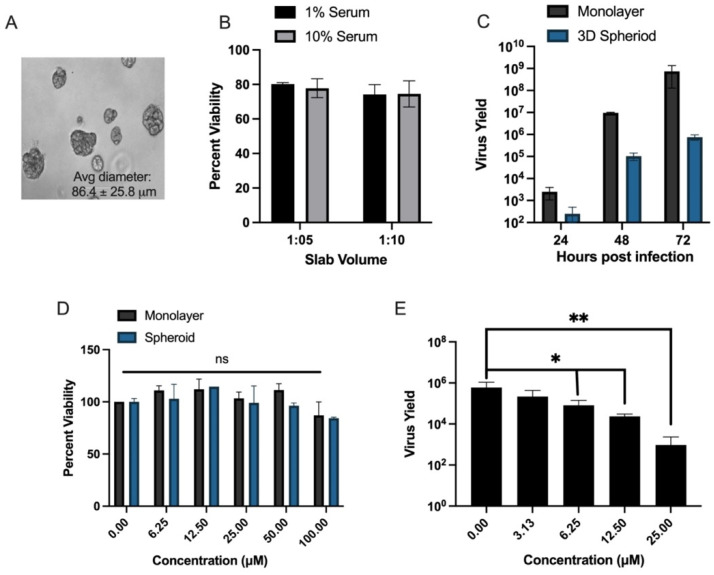
Evaluation of a 3D human hepatoma spheroid model for YFV infection and antiviral compound activity. Huh-7 cells were plated in a 1:5 or 1:10 suspension of media: Matrigel^®^ in 1% or 10% serum and grown for 3 weeks. (**A**) Microscopic analysis of 3D cultures at 14 days post-seeding. (**B**) Cellular viability after 14 days in culture as determined by trypan blue dye exclusion assay; shown mean ± SEM, *n* = 12. (**C**) Kinetics of YFV replication in 2D and 3D spheroid Huh-7 cultures. 10^3^ cells were seeded in a 96-well plate and grown for 1 day (2D) or 2 weeks (3D spheroids) then infected with YFV (MOI 1). At 24, 48, and 72 h post-infection (hpi) cultures were collected, total RNA isolated, and YFV genome copies detected by qPCR. (**D**) MTS assay to assess cytotoxicity of C9 in 2D and 3D spheroid cultures. The 10^3^ cells were seeded in a 96-well plate and grown for 1 day (2D) or 2 weeks (3D spheroids) then treated with increasing amounts of compound (0–100 µM), left untreated (positive control) or treated with 0–10 µM of cycloheximide (experimental control; data not shown). After 4 days, cultures were assayed for viability by their ability to reduce MTS over 4 h. (**E**) Antiviral activity of C9 in YFV-infected 3D spheroid cultures. The 10^3^ cells were seeded in a 96-well plate and grown for 2 weeks then treated with 0–25 µM of C9 and infected with YFV MOI 1.0 for 3 days. At 72 hpi, cultures were collected, total RNA isolated, and YFV genome copies detected by qPCR. (**C**,**E**) Relative genome copy numbers were correlated to virus yield via a standard curve. EC_50_ and EC_90_ values were calculated by linear regression. (**D**,**E**) Reduction in viability (**D**) and viral load (**E**) was determined by one-way ANOVA in GraphPad Prism v9. Data shown as mean ± SEM, *n*  =  6. *p*-values * (6.25 µM = 0.0244; 12.5 µM = 0.0124) ** = 0.0095.

3D cultures provide a novel, highly biomimetic microenvironment for the study of compound efficacy [33,34]. As such, we developed a 3D hepatocyte spheroid model derived from the Huh-7 cell line to assay C9′s cytotoxicity and antiviral activity. Culture conditions were optimized to seeding in a 1:5 slab of media: Matrigel^®^ with 1% serum as these parameters produced spheroids of optimal size (avg diameter 86.4 ± 25.8 µm; Figure 4A) and >80% viability over 3 weeks in culture (Figure 4B). This diameter is ideal for long-term viability and to enhance nutrient and drug uptake, issues common to 3D culture [33]. The utility of these spheroids for the study of YFV was validated by performing virus kinetics in 2D and 3D cultures over 3 days at MOI 1.0. YFV readily infected and replicated in 3D spheroids with notable CPE and virus output ~3 logs over input (Figure 4C). To evaluate changes in compound C9′s toxicity in the 3D model, spheroids were treated with increasing amounts of C9 (0–100 µM) for 4 days, then assayed for the ability to reduce MTS. At 100 µM, viability dropped to 84.3 ± 0.9% (Figure 4D). This value was higher than what was seen in 2D cultures with a CC_50_ of 68.5 µM (Table 1). To account for assay differnces, C9 was reevaluated in 2D following the same methodology for 3D cultures and exhibited a similar 87.1% viability at 100 µM (data not shown). Antiviral dose-response assays were next performed by infecting with YFV (MOI 1.0) in the presence or absence of increasing concentrations (0–25 µM) of C9 (Figure 4E). As expected, the effective concentration of C9 was slightly higher in 3D cultures compared with 2D cultures (EC_50_ 3.2 versus 0.85 µM, respectively; Figure 4E).

**Figure 5:**
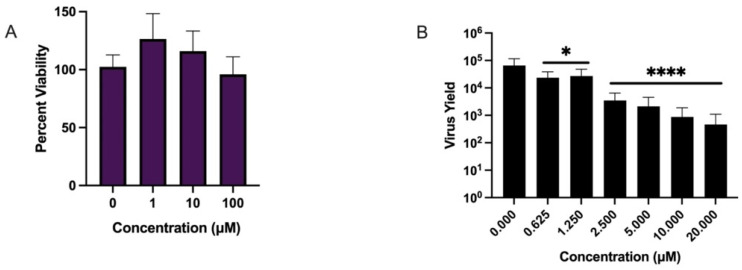
Cytotoxicity and anti-YFV activity of C9 in primary human macrophages. Monocytes were isolated from human blood (pooled from three donors), plated at 10^5^ cells per well in a 96-well plate, and treated with GM-CSF for 10 days. (**A**) MTS reduction assay. MDMs were treated with increasing amounts of compound (0–100 µM), left untreated (positive control), or treated with 0–10 µM of cycloheximide (experimental control) for 4 days. After 4 days, cultures were assayed for viability via ability to reduce MTS over 4 h. Reduction in viability was determined by one-way ANOVA with Dunnet’s correction for multiple comparisons in GraphPad Prism v9. Data shown as mean ± SEM, *n*  =  6. (**B**) Antiviral activity of C9 in YFV-infected MDM cultures. Cells were treated with 0–10 µM of C9 and infected with YFV MOI 1.0 for 3 days. At 72 hpi, supernatants were collected, total RNA isolated, and YFV genome copies detected by qPCR. Relative genome copy numbers were correlated to total virus yield via a standard curve. EC_50_ and EC_90_ values were calculated by linear regression and the significance of reduction by one-way ANOVA with Dunnet’s correction for multiple comparisons in GraphPad Prism v9. Data shown as mean ± SEM, *n*  =  8. *p*-value * (0.625 µM = 0.01; 1.25 µM = 0.016), **** ≤ 0.0001.

Macrophages are important permissive cells that support YFV replication and constitute a viral reservoir in pathogenesis [5]. Thus, we chose to evaluate C9′s efficacy and activity in primary human macrophages (MDMs). MDM cultures were established from monocytes isolated from human blood (NY Blood Bank, New York, NY, USA), then differentiated with GM-CSF for a period of 10 days post-expansion [15,16]. Cytotoxicity was evaluated by MTS assay (Figure 5A). C9 had a slight stimulatory effect at low (1 µM) concentrations with no notable cytotoxicity up to 100 µM (Figure 5A). To assess antiviral activity, MDMs were infected with YFV (MOI 1.0) in the presence or absence of increasing concentrations of compounds (0–20 µM) (Figure 5B). C9 significantly reduced YFV replication in MDM with EC_50_ 0.4 ± 0.1 µM and EC_90_ 1.5 ± 0.6 µM (Figure 5B) with comparable efficacy as sofosbuvir (EC_50_ 0.4 ± 0.3 µM; data not shown). Collectively, the data generated from 3D heptoma spheroids and primary human macrophages confirmed the low cytotoxicity and potent anti-YFV activity of compound C9 and justified further evaluation in vivo.

### 3.5. In Vivo Anti-YFV Activity of C9 in A129 Mice

**Figure 6:**
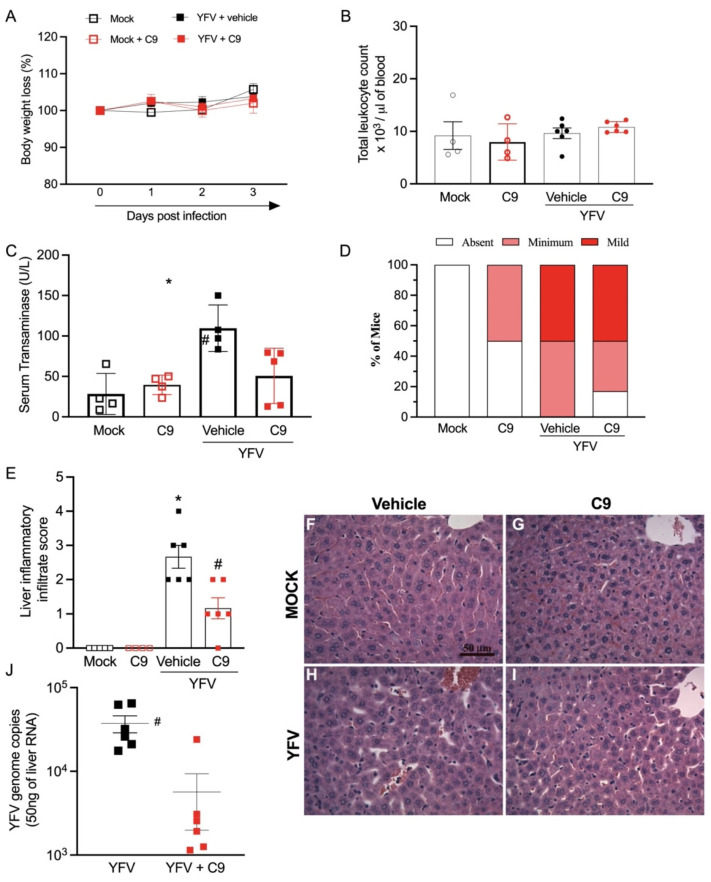
In vivo anti-YFV activity of C9 in A129 mice. Adult (8–10 weeks old) A129 mice were infected i.v. with 10^6^ PFU/100 μL of YFV virus (YFV_17D_ strain). Daily intraperitoneal injections of C9 (10 mg/kg) were started 1 hr post-infection and continued until 3 days post-infection (dpi). Mice were euthanized 6 hr after the last treatment with vehicle or C9 on day 3 post-infection. Blood and tissues were collected and processed for further analysis. Groups were composed of six mice, three males, and three females. (**A**) Body weight change post-infection was assessed by two-way ANOVA plus Sidak’s multiple-comparison test. Data are shown as mean ± SEM, *n*  =  6. (**B**) Changes in the number of circulating total leukocytes 3 days post-inoculation, represented as box plots. Data from each group were compared with those from the mock or mock + C9 groups by one-way ANOVA plus Dunnett’s multiple-comparison test data shown as mean ± SEM, *n*  =  6. (**C**) Estimation of liver dysfunction analyzed by serum concentration of alanine aminotransferase (ALT). Results are expressed as U/L. Statistical differences among groups were assessed by one-way ANOVA plus Tukey’s post hoc test data shown as mean ± SEM, *n*  =  6. (**D**) Percentages of mice according to the grade of inflammatory cell infiltration in the liver. (**E**) Histopathological assessment in relation to overall inflammatory score. Comparisons between mock and mock + C9 and infection groups were carried out by Kruskal-Wallis plus Dunn’s post hoc test data shown as mean ± SEM, *n*  =  6. (**F**–**I**) Representative H and E-stained liver slices of mock (**F**), mock + C9 treated (**G**), YFV + vehicle (**H**), and YFV + C9-treated mice (**I**) at 3 dpi. Scale bar: 100 µm. (**J**) Quantitative RT-PCR analysis of YFV viral load in the mice livesr tissue 3 days after YFV inoculation. Data shown as mean ± SEM, *n*  =  6; * *p* < 0.01 in comparison to the non-infected (mock) group,; # *p* < 0.01 when comparing YFV + C9 to YFV group using unpaired *t* test.

Based on the data generated in 2D and 3D model systems, in vivo studies to assess compound C9′s anti-YFV activity were performed in interferon receptor-deficient A129 mice, a widely used model for YFV studies [35,36]. Equal numbers of male and female A129 mice were infected with 10^6^ PFU of YFV 17D vaccine strain, then treated with 10 mg/kg of C9, or saline vehicle control, through intraperitoneal injection 1 hr after and on days 1, 2, and 3 post-infection (dpi). At 3 dpi, 6 hr after the last C9 administration, mice were euthanized. Weight loss was measured daily and revealed no significant difference between control or test groups, indicative of a mild infection associated with strain 17D during the first week of YFV infection [35,36]. Administration of C9 had no adverse impact on weight loss or total circulating leukocytes (Figure 6A,B). YFV infection significantly elevated levels of serum transaminase (ALT; *p* = 0.014; Figure 6C). Histopathologic examination of liver sections revealed that YFV 17D infection triggered transient inflammation-associated liver injury. As such, approximately 50% of vehicle-treated mice had mild and 50% minimum inflammatory cell infiltration while C9-treated mice showed 50% mild, 25% minimum, and 25% absent (Figure 6D). Immune cell infiltration into the liver was significantly reduced (*p* < 0.0001) in C9-treated mice in comparison with vehicle-treated littermates (Figure 6D). Panels F-I are representative of Figure 6E. Finally, PCR analysis of YFV genome copy levels also showed a significant reduction in virus load in infected mice receiving C9 treatment (*p* = 0.0067; Figure 6J). These data indicate that compound C9 had potent anti-YFV activity when administered after infection in vivo without notable adverse effects during the early stages of infection in A129 mice.

## 4. Discussion

Numerous C2′-sugar-modified ribonucleosides with demonstrated antiviral activity have been designed against the well-studied flavivirus HCV [12,13,37]. There is a high degree of structural similarity in the ligand-binding pocket of the RdRps of both HCV and YFV [9] and computer modeling predicts sofosbuvir exhibits comparable binding affinity for the YFV RdRp model built from the 3-D structure of HCV RdRp [9]. Thus, we evaluated similarly designed compounds as potential anti-YFV agents. Based on our identification of the 2′-halo nucleoside analogs sofosbuvir (C1) and 2′-dichlorouridine (C3) [11] as YFV inhibitors, we extended our study by evaluating other 2′-dihalo-uridine nucleoside analogs and their respective phosphoramidate prodrugs as potential anti-YFV agents.

Compounds 1-11 were evaluated for anti-YFV activity in human hepatomas (Huh-7s), a primary target cell for YFV. Of the 11 initial compounds screened, the 2′-dichloro- (C3), 2′-chloro,2′-fluoro- (C5) and the 2′-bromo,2′-fluoro- (C9) nucleoside prodrugs displayed sub-micromolar anti-YFV activity (Table 1). Of note, C3 had the lowest EC_90_ value of 3.2 ± 2.7 µM; however, this was not significantly different than that of C5 (6.5 ± 2.6 µM) or C9 (7.8 ± 3.6 µM). To establish a toxicity profile, all compounds were evaluated for cytotoxicity in Huh-7s, Vero, PBM, and CEM cells. Prodrugs C 1, 5, 7, 9, and 11 exhibited cytotoxicity below 100 µM in the Huh-7 cells only. Compounds C 7 and 11 had significant toxicity in this cell line (2.2 ± 0.9 µM and 8.7 ± 1.0 µM, respectively). Prodrugs C5 and C9 had CC_50_ values greater than 45 µM, which, given their sub-micromolar anti-YFV activity, provides an ample therapeutic window for their development. Owing to previously established favorable toxicity and in vivo pharmacokinetic profiles in human liver and intestinal microsomes as well as male beagles [13] and the development of a new process chemistry method by our group for large-scale synthesis [38], we selected C9 as a candidate for further evaluation.

To better understand the binding of 2′-halo nucleoside analogs of sofosbuvir (C1), we predicted and compared the binding of our lead 2′-fluoro,2′-bromouridine triphosphate (C8-TP) with 2′-fluoro,2′-methyluridine-5′ triphosphate (Figure 3). C8-TP had ~25% higher binding affinity for the YFV RdRp compared to 2′-fluoro,2′-methyluridine-5′ triphosphate. Previously, molecular modeling predicted the binding mode of 2′-fluoro,2′-methyluridine-5′ triphosphate with YFV RdRp using a homology model built from HCV RdRp. In contrast, we used the recently released crystal structure of YFV RdRp. Our predicted binding mode of 2′-fluoro,2′-methyluridine-5′ triphosphate, and C8-TP with YFV RdRp had interactions or in proximity with catalytic residues D541, D535, or S603 which were not observed in previous models. Thus, our model should be more reliable and accurate. Multiple sequence alignment of the YFV RdRp catalytic domain from 16 different isolates revealed an overall >98% homology in the region and, importantly, conservation of residues involved in binding to C9. Overall, our model predicts that our lead compound C9, in active NTP form, will readily bind to the active site of the YFV RdRp which should confer its anti-YFV activity. Additional biochemical studies on the mechanism of action of nucleoside analogs on the viral polymerase are ongoing.

The inclusion of 3D cell culture models in the drug pipeline has greatly reduced the time and the costs of moving from the lab to clinical studies primarily as 3D models, bearing higher physiological relevance, are expected to be most indicative of both cytotoxicity and in vivo activity. Thus, we developed in-house a 3D Matrigel^®^-embedded hepatoma spheroid model for further investigation of C9 before moving into an animal model. The 3D spheroids were viable long-term (≥14 days; Figure 4B) and readily supported YFV infection and replication (Figure 4C). Given the differences in metabolic pathways noted throughout the literature from 3D to 2D models, we assessed the cytotoxicity of C9 in both systems. When experimental conditions were adjusted to match both systems, cytotoxicity was similar at >80% at 100 µM (Figure 4D). C9 exhibited anti-YFV activity with an EC_50_ 3.2 µM in 3D spheroids (Figure 4E) compared to EC_50_ 0.9 µM in 2D cultures (Table 1). Differences in a drug’s effective concentration in 2D vs. 3D is a common phenomenon as spheroids have different and more ‘in-vivo-like’ biochemical properties than flat monolayer cultures. As such, EC_50/90_ values from 3D cultures are generally considered to be more indicative of in vivo activity.

Macrophages also play an important role in YFV infection as they serve as a virus reservoir as well as facilitating virion transport to other tissues/organs [5]. C9 had potent anti-YFV activity in MDMs with effective concentrations even lower than seen in Huh-7s (EC_50_ 0.4 vs. 0.9 µM; EC_90_ 1.5 vs. 7.8 µM respectively; Table 1, Figure 5B) and comparable to sofosbuvir (EC_50_ 0.4 ± 0.3 µM; data not shown). Antiviral data generated in MDMs provide additional value as this is the first account, to our knowledge, of nucleoside analogs with potent anti-YFV activity in human macrophages. The ability of C9 to target and reduce YFV replication in both circulating macrophages as well as target hepatocytes has strong implications for the potential therapeutic value of C9 as a new anti-YFV agent.

Compound C9′s efficacy was tested in the murine model against the YFV 17D vaccine strain. In our experiments, we chose to evaluate mice three days after infection to directly assess the impact of C9 on early stages of infection. A 10 mg/kg dosage was selected as this has proven effective with other nucleoside analogs in previous murine anti-flavivirus studies [39,40]. YFV 17D presents with self-limiting disease and mild pathology in A129 mice during the first week of infection [35,36] as was evidenced by no mortality or significant change in body weight or total leukocyte counts post-infection (Figure 6A,B). Administration of C9 also had no significant impact on these parameters, suggesting no adverse effect on mice (Figure 6A,B). Importantly, C9 offered significant protection against YFV-induced liver damage and inflammation as well as significantly reduced viral burden in the liver (Figure 6C–J). To our knowledge, sofosbuvir is the only other NA to have been tested as an anti-YFV agent in mice [10]. In the 2019 study, sofosbuvir failed to reduce viral burden in the sera, livers, kidneys, and spleens of YFV-infected A129 mice [10]. Pre-treatment with sofosbuvir afforded protection from long-term loss in body weight, liver damage, and viral burden in the brain suggesting that sofosbuvir may be offered as a prophylactic treatment but could have reduced efficacy given therapeutically [10]. Recently, Mendas et al. reported the use of sofosbuvir on a compassionate basis in two women hospitalized with YFV infection and while they were eventually able to clear the infection this was not a controlled study [9]. In our mouse study, C9 administration was very effective and our data herein warrant its further development as a YFV antiviral agent.

In conclusion, we demonstrated the efficacy of a 2′-fluoro,2′-bromouridine prodrug (C9) as an effective anti-YFV agent in both hepatomas and macrophages as well as in the A129 mouse model. Our previous studies on C9 show that this compound exhibits a highly favorable toxicity profile and excellent pharmacokinetics with potent anti-HCV activity [13] and can be manufactured on a large scale and with a high yield [38]. For the first time, we have shown that C9 also has a pronounced antiviral and anti-inflammatory effect on YFV in an animal model when administered post-infection. Our data lend support for the further preclinical evaluation of C9 as a therapeutic option for YFV infections. The successful discovery and development of a YFV antiviral has potentially far-reaching impact and opportunity to curb future epidemics by limiting spread and morbidity.

## Figures and Tables

**Table 1:** Antiviral Activity and Cytotoxicity of Compounds 1-11.

Compound	Anti-YFV Activity ^a^ (Huh-7)		Cytotoxicity ^b^: CC_50_ (µM) ^c,e^			
	EC_50_ (µM) ^d^	EC_90_ (µM) ^d^	PBM	CEM	Vero	Huh-7
Sofosbuvir 1	0.6 ± 0.6	3.5 ± 1.5	>100	>100	>100	88.5 ± 11.6
2	>10	>10	>100	>100	>100	>100
3	0.4 ± 0.5	3.2 ± 2.7	>100	>100	>100	>100
4	>10	>10	>100	>100	>100	>100
5	0.4 ± 0.2	6.5 ± 2.6	>100	>100	>100	46.7 ± 13.3
6	>10	>10	>100	>100	>100	>100
7	>10	>10	>100	>100	>100	2.2 ± 0.9
8	>10	>10	>100	>100	>100	>100
9	0.9 ± 0.8	7.8 ± 3.6	>100	>100	>100	68.9 ± 6.3
10	>10	>10	>100	>100	>100	>100
11	>10	>10	>100	>100	>100	8.7 ± 1.0

^a^ Detection of YFV genomes in infected cell supernatants by qRT-PCR. ^b^ Determination of compound cytotoxicity by MTS assay. ^c^ CC_50_ = concentration displaying 50% cytotoxicity compared with untreated. ^d^ EC = 50% (EC_50_) or 90% (EC_90_) effective antiviral concentration. ^e^ PBM = human peripheral blood mononuclear cells. CEM = human T lymphoblasts. Vero = African Green Monkey kidney epithelial cells. Huh-7 = human hepatoma cells.
